# SARS-CoV-2 Testing Service Preferences of Adults in the United States: Discrete Choice Experiment

**DOI:** 10.2196/25546

**Published:** 2020-12-31

**Authors:** Rebecca Zimba, Sarah Kulkarni, Amanda Berry, William You, Chloe Mirzayi, Drew Westmoreland, Angela Parcesepe, Levi Waldron, Madhura Rane, Shivani Kochhar, McKaylee Robertson, Andrew Maroko, Christian Grov, Denis Nash

**Affiliations:** 1 Institute for Implementation Science in Population Health City University of New York New York, NY United States; 2 The Carolina Population Center University of North Carolina at Chapel Hill Chapel Hill, NC United States; 3 Department of Maternal and Child Health Gillings School of Global Public Health University of North Carolina at Chapel Hill Chapel Hill, NC United States; 4 Department of Epidemiology and Biostatistics Graduate School of Public Health and Health Policy City University of New York New York, NY United States; 5 Department of Environmental, Occupational, and Geospatial Health Sciences Graduate School of Public Health and Health Policy City University of New York New York, NY United States; 6 Department of Community Health and Social Sciences Graduate School of Public Health and Health Policy City University of New York New York, NY United States

**Keywords:** COVID-19, SARS-CoV-2, discrete choice experiment, implementation science, engagement, testing, cohort study, stated preference study, pandemic

## Abstract

**Background:**

Ascertaining preferences for SARS-CoV-2 testing and incorporating findings into the design and implementation of strategies for delivering testing services may enhance testing uptake and engagement, a prerequisite to reducing onward transmission.

**Objective:**

This study aims to determine important drivers of decisions to obtain a SARS-CoV-2 test in the context of increasing community transmission.

**Methods:**

We used a discrete choice experiment to assess preferences for SARS-CoV-2 test type, specimen type, testing venue, and results turnaround time. Participants (n=4793) from the US national longitudinal Communities, Households and SARS-CoV-2 Epidemiology (CHASING) COVID Cohort Study completed our online survey from July 30 to September 8, 2020. We estimated the relative importance of testing method attributes and part-worth utilities of attribute levels, and simulated the uptake of an optimized testing scenario relative to the current typical testing scenario of polymerase chain reaction (PCR) via nasopharyngeal swab in a provider’s office or urgent care clinic with results in >5 days.

**Results:**

Test result turnaround time had the highest relative importance (30.4%), followed by test type (28.3%), specimen type (26.2%), and venue (15.0%). In simulations, immediate or same-day test results, both PCR and serology, or oral specimens substantially increased testing uptake over the current typical testing option. Simulated uptake of a hypothetical testing scenario of PCR and serology via a saliva sample at a pharmacy with same-day results was 97.7%, compared to 0.6% for the current typical testing scenario, with 1.8% opting for no test.

**Conclusions:**

Testing strategies that offer both PCR and serology with noninvasive methods and rapid turnaround time would likely have the most uptake and engagement among residents in communities with increasing community transmission of SARS-CoV-2.

## Introduction

The Centers for Disease Control and Prevention recently estimated that for every case of SARS-CoV-2 infection diagnosed in the United States, an additional 10 are undiagnosed [1]. Detecting a higher proportion of people with active infection via widespread testing is a prerequisite to achieving the public health goals of controlling the transmission of SARS-CoV-2 [2,3]. However, limited access to and uptake of testing for many in the United States, combined with lengthy result turnaround time, severely hampers pandemic control efforts, which require timely detection, isolation, and quarantine. Although recent increases in testing are promising [4], some models [5] suggest a shortfall, and important populations may still be unreached [6]. Understanding factors that may influence an individual’s decision to seek testing can help enhance and sustain uptake of SARS-CoV-2 testing when, where, and among whom it is needed most for public health purposes. These factors include individual preferences for different types of testing services, which have not been systematically ascertained or incorporated into testing service delivery.

## Methods

To identify the most preferred SARS-CoV-2 testing scenarios for individuals, we conducted a discrete choice experiment (DCE) [7,8] in a US national longitudinal cohort of adults being followed for SARS-CoV-2 seroconversion and other related outcomes. DCEs are a powerful tool to identify the most preferred attributes in populations being targeted for health interventions and can inform strategies to increase interventions’ uptake and engagement.

### Study Population

We invited all participants of the Communities, Households and SARS-CoV-2 Epidemiology (CHASING) COVID Cohort Study [9] who completed a recent routine follow-up assessment (n=5098) to participate in the DCE. CHASING COVID Cohort Study participants were recruited online using internet-based strategies, including via referral, social media advertisements in English and Spanish, and Qualtrics Panel [9]. Recruitment and advertising strategies were periodically adjusted to increase diversity across racial, ethnic, and age groups. Eligibility criteria included being 18 years or older and residing in the United States, Puerto Rico, or Guam at enrollment. Participants provided informed consent at the baseline assessment and separately for SARS-CoV-2 antibody testing. A total of 4793 (94% of those invited) completed the DCE July 30 to September 8, 2020. A US $5 Amazon gift card incentive was offered to participants completing the DCE.

### DCE Design, Analysis, and Simulation

The DCE was designed and implemented using Lighthouse Studio 9.8.1 (Sawtooth Software) and deployed using Sawtooth’s online survey hosting platform. Participants were asked to consider different combinations of SARS-CoV-2 testing service features in a situation where “...the number of people hospitalized or dying from coronavirus in your community was increasing.” Each participant was presented with five choice tasks, each containing two juxtaposed scenarios comprised of different combinations of the testing features (aka attribute levels) and a “None” option if neither testing scenario was appealing or desirable. Testing service attributes included in the DCE are shown in Table 1 and included: type of test, specimen type, testing venue, and results turnaround time (see also Multimedia Appendix 1). The combinations presented and the order of their presentation to each participant were randomized to reduce bias (see Multimedia Appendix 2).

We estimated zero-centered part-worth utilities for each attribute level and overall relative attribute importance using effects coding in a hierarchical Bayesian model [10]. We used these estimates to simulate changes in uptake of the different testing scenarios that resulted from “swapping” each individual attribute level in Table 1 into the current typical testing option of a polymerase chain reaction (PCR) test using a nasopharyngeal (NP) swab in a doctor’s office or urgent care clinic, with results returned in >5 days. The baseline simulation contained three scenarios: (1) the primary current typical testing scenario; (2) a second, duplicate current typical testing scenario; and (3) a no test scenario. Each attribute level was then individually varied in the duplicated scenario, holding all levels in the other attributes in the duplicated scenario constant. The uptake of each varied scenario was simulated along with the two other original scenarios, and the uptake of the modified scenario was compared to the uptake of the primary current typical testing scenario in the baseline simulation.

We also created a hypothetical testing scenario that optimized preferences across attributes, which included PCR and serology from a saliva sample collected at a pharmacy with same-day results. We then simulated the proportion of participants who would choose this optimized scenario, the current typical testing option, or neither option. For all simulations, predicted uptake of each testing strategy was estimated using the randomized first choice method [11,12], which computes the proportion of participants that would choose each testing scenario based on its total utility, over thousands of draws per participant, assuming that each participant would select the scenario that provides them with the highest total utility summed across attributes. DCE data were analyzed and simulations were conducted using Lighthouse Studio 9.8.1.

**Table 1:** SARS-CoV-2 testing discrete choice experiment attributes and levels.

Attributes and levels (abbreviated)^a^		Descriptive-level text^b^
**Test**		
	Serology	An antibody test that tells you if you've EVER had a COVID-19 infection
	PCR^c^	A PCR test that tells you if you CURRENTLY have a COVID-19 infection
	Both tests	BOTH an antibody test (EVER infected) and a PCR test (CURRENTLY infected)
**Specimen type**		
	Finger prick	A small amount of blood from a finger prick
	Blood draw	A small tube of blood taken from your arm
	Cheek	Oral fluid from a swab of the inside of your cheek
	Spit	A spit sample collected in a small cup
	Nasal shallow	A SHALLOW swab of the inside of your nostrils
	NP^d^ swab	A DEEP swab that goes far into your nasal passages
	Urine	A urine sample collected in a small cup
**Venue**		
	Home collection, receiving, and returning kit in mail	You are mailed a package with the test kit; you collect the specimen and mail it back to the lab
	Home collection, receiving kit in mail, and returning to a collection site	You are mailed a package with the test kit; you collect the specimen and drop it off at a collection site near your home
	Doctor’s office or urgent care clinic	You go to your doctor's office or an urgent care clinic to have the specimen collected
	Walk-in community testing site	You go to a walk-in community testing site to have the specimen collected
	Drive-through community testing site	You go to a drive-through community testing site to have the specimen collected (you stay in your car)
	Pharmacy	You go to a local pharmacy to have the specimen collected
**Results turnaround time**		
	Immediate	Immediately (within 15 minutes)
	Same day	On the same day
	48 hours	Within 48 hours
	5 days	Within 5 days
	Greater than 5 days	>5 days

^a^Some combinations of attribute levels were prohibited. For example, a test scenario that included a specimen collected at home and returned to the lab via mail could not also include the immediate test result level.

^b^Descriptive text was displayed in the choice exercise.

^c^PCR: polymerase chain reaction.

^d^NP: nasopharyngeal.

### Ethical Review

This study was approved by the Institutional Review Board at the City University of New York (CUNY) Graduate School of Public Health.

## Results

### Participant Demographic Characteristics

Participants’ median age was 39 (IQR 30-53) years. Out of the 4793 participants, 51.5% (n=2468) identified as female, 45.8% (n=2193) identified as male, and 2.8% (n=132) identified as other gender identities (nonbinary, transgender male, transgender female). Additionally, 62.8% (n=3009) identified as non-Hispanic White, 16.4% (n=788) identified as Hispanic, 9.2% (n=442) identified as non-Hispanic Black, 7.5% (n=361) identified as Asian, 3.9% (n=189) identified as another race or ethnicity, and 0.1% (n=4) had missing information for race and ethnicity. At enrollment, 29.0% (n=1391) of participants resided in the Northeast, 28.3% (n=1358) resided in the South, 23.9% (n=1146) resided in the West, 17.7% (n=850) resided in the Midwest, and 0.2% (n=7) resided in Puerto Rico or Guam; 0.9% (n=41) of participants affirmed US residence but did not provide a zip code with which to assign them a region.

### Relative Importance of Testing Service Attributes and Attribute Levels

Results turnaround time had the highest relative importance (30.4%), followed by test type (28.3%), specimen type (26.2%), and venue (15.0%; see Table S1 in Multimedia Appendix 3). Participants strongly preferred rapid receipt of results, with progressively weaker preference for slower test results. Within test type, participants showed a strong preference for testing scenarios that detect both current and past infection (see Table S2 in Multimedia Appendix 3). Participants most preferred testing scenarios that use cheek swab specimens and least preferred scenarios that require a deep NP swab. There was a preference for at-home self-collection of specimens using kits received and returned via mail; testing in a doctor’s office or urgent care clinic was the least preferred testing venue. Participants chose neither testing option in only 3.6% (861/23,965) of the choice tasks.

### Simulation Results

**Figure 1:**
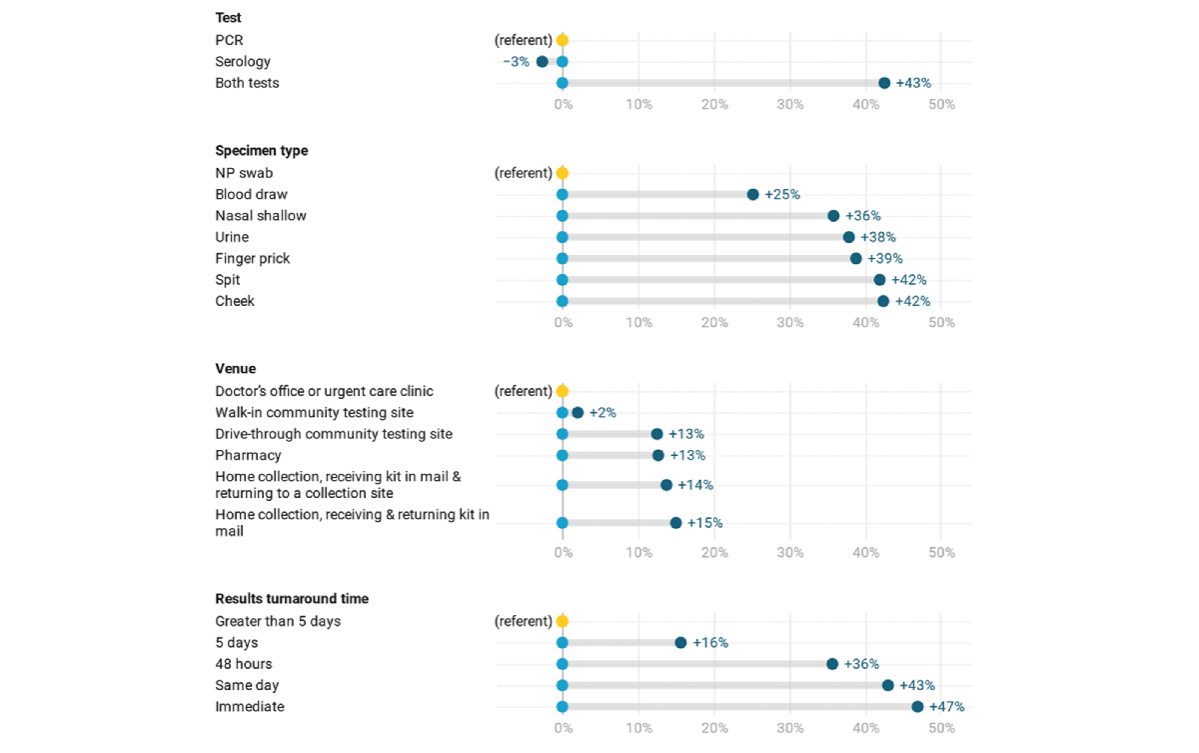
Simulated changes in SARS-CoV-2 testing uptake relative to the current typical testing option, by attribute level. The current typical testing option is PCR via NP swab in a provider’s office or urgent care clinic with results in >5 days. The baseline simulation included the current typical testing option compared to a second duplicate current typical testing scenario and a no test scenario. Changes in uptake in subsequent simulations were estimated by individually varying each attribute level in the duplicated scenario, holding other attributes constant. The referent value of zero is the difference between the original and duplicated typical testing scenarios at baseline. NP: nasopharyngeal; PCR: polymerase chain reaction.

Simulating changes in SARS-CoV-2 testing uptake by varying attribute levels individually, we found the largest marginal increases in testing uptake from including immediate test results (+47%) or same-day test results (+43%), with more modest increases for results 48 hours after the test (+36%) and 5 days after the test (+16%), compared to results in >5 days (see Figure 1). Testing scenarios that offered both PCR and serology also substantially increased marginal uptake (+43%), whereas serology testing alone slightly decreased uptake (–3%). Among specimen types, oral specimens (cheek or spit [+42%]) had the largest increase in uptake over NP swab, followed by finger prick (+39%), urine (+38%), shallow nasal swab (+36%), and blood draw (+25%). Though smaller in magnitude, we found increases in uptake for testing venue alternatives to a doctor’s office or urgent care clinic, with the greatest increases for the home testing venues (receiving and returning the test kit in the mail [+15%] and receiving the kit in the mail and returning to a collection site [+14%]), followed by pharmacy (+13%), drive-through community testing site (+13%), and walk-in community testing site (+2%).

We also simulated the proportion of participants that would pick the current typical testing scenario versus a scenario with multiple more preferable features: both PCR and serology using a saliva specimen collected at a pharmacy with same-day test results. Simulated uptake of this hypothetical scenario was 97.7% compared to 0.6% for the current typical testing scenario, with 1.8% opting for no test when presented with these two choices.

## Discussion

### Principal Results

Our participants preferred faster test results from less invasive specimens collected at home that provide comprehensive information about current and past infection. From a public health perspective, faster test results are more actionable [13], and at the individual level, delayed test results can provoke anxiety in other diagnostic settings [14-16]. Participants tended to favor specimen collection venues that could be construed as more convenient (pharmacy or drive-through testing site) or better able to facilitate social distancing (home), compared to a walk-in clinic or doctor’s office, where one might be more likely to come into contact with infectious individuals. Our venue-related results are in line with findings from the HIV and sexually transmitted infection literature, where at-home specimen collection for diagnostic testing has high acceptability and reliability [17,18], and with other recent findings indicating high willingness to collect at-home specimens for a SARS-CoV-2 research study [19]. The strong preference for both PCR and serology may be related to a belief that antibodies confer immunity against subsequent infection [20,21] and a general desire to get the most utility out of a single specimen.

Our findings suggest that expected advances in SARS-CoV-2 testing technologies, such as rapid, at-home saliva tests, will be highly acceptable and used when they become available, particularly in communities with increasing deaths or hospitalizations. Some preferred tests for SARS-CoV-2 (eg, at-home rapid antigen tests) may be less sensitive than gold standard diagnostic tests (PCR via NP swab). Nevertheless, these findings are significant from a public health standpoint since it’s possible that widespread and frequent use of a less sensitive SARS-CoV-2 antigen test could detect much greater numbers of people with active infection—and more quickly—than the current typical testing scenario [22]. Indeed, our data suggest that NP swabs may be a deterrent to testing, which could be addressed by adding serology or relying on saliva specimens.

### Limitations

Limitations of this study include the omission of other attributes that may influence testing preferences, such as frequency of testing, cost, facility wait times, or distance. In addition, the majority of our participants had already completed at-home self-collection of a dried blood spot specimen for our study. Though the venue attribute had the lowest relative importance, this prior experience may have influenced their preferences for venue in the DCE.

### Conclusions

To the extent that it is possible to align public health strategies to deliver testing services with the preferences of those being targeted for testing, greater uptake and engagement may be achieved. Additional research is needed to increase SARS-CoV-2 testing uptake in ways that are aligned with the public health goals of the pandemic response, including preferences for engaging in public health interventions following a positive test, such as isolation and contact tracing [3].

## Appendix

Multimedia Appendix 1 Desktop example of SARS-CoV-2 testing preferences choice task.

Multimedia Appendix 2 Supplementary methods.

Multimedia Appendix 3 Table S1 (average relative attribute importance for SARS-CoV-2 testing features) and Table S2 (part-worth utilities for SARS-CoV-2 testing features).

## Abbreviations

CHASING: Communities, Households and SARS-CoV-2 Epidemiology
CUNY: City University of New York
DCE: discrete choice experiment
NP: nasopharyngeal
PCR: polymerase chain reaction
