# Supplementary material for: SARS-CoV-2 Testing Service Preferences of Adults in the United States: Discrete Choice Experiment
Source: JMIR Public Health Surveill. 2020 Dec 31;6(4):e25546. doi: 10.2196/25546 (PMC7781587; doi:10.2196/25546)
Supplement: Multimedia Appendix 1 [file publichealth_v6i4e25546_app1.docx]

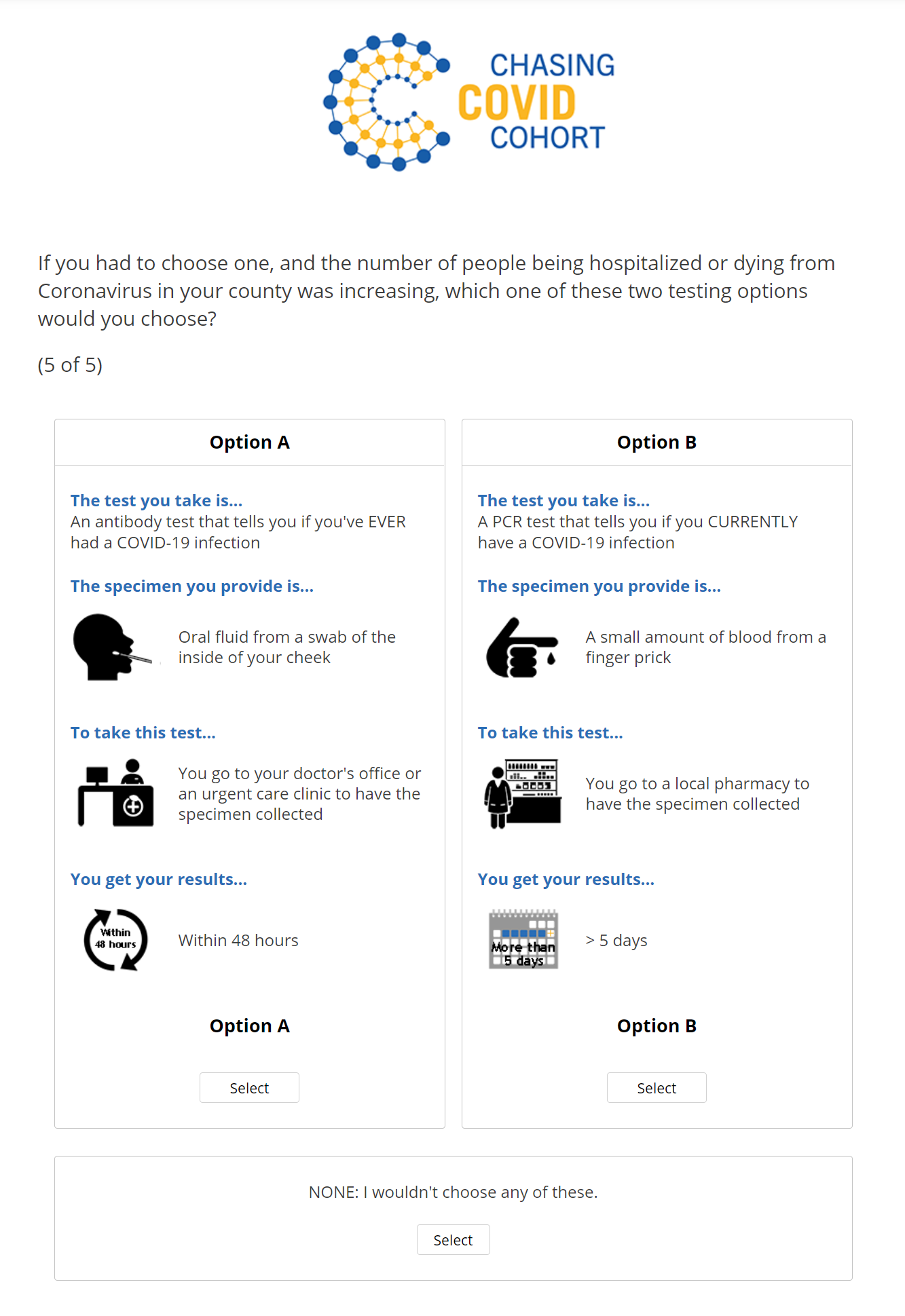


*Supplementary Figure 1****.*** *Desktop example of SARS-CoV-2 testing preferences choice task.* Designed and implemented using Sawtooth Lighthouse Studio 9.8.1 (Sawtooth Software, Provo, UT). Images from The Noun Project.
