# Supplementary material for: SARS-CoV-2 Testing Service Preferences of Adults in the United States: Discrete Choice Experiment
Source: JMIR Public Health Surveill. 2020 Dec 31;6(4):e25546. doi: 10.2196/25546 (PMC7781587; doi:10.2196/25546)
Supplement: Multimedia Appendix 2 [file publichealth_v6i4e25546_app2.docx]

**Supplementary Methods:** The final DCE design included 500 survey versions in which each level appeared approximately the same number of times as the other levels within each attribute across the five tasks, some level overlap within an attribute was permitted across concepts in the same task, and levels within one attribute were included independently of levels within other attributes—Sawtooth’s Balanced Overlap design.[1,2] The design was tested with 2500 dummy participants and assuming “None” was chosen in 33% of choice tasks, yielding estimated standard errors ranging from 0.0212 to 0.0489, with an absolute D-efficiency of 1342.39. For comparison, in a completely enumerated design, also with 2500 dummy participants and 33% choosing “None,” the estimated standard errors ranged from 0.0208 to 0.0499 with an absolute D-efficiency of 1380.008; our design’s relative D-efficiency was 97%.

References

[1] Sawtooth Software. Lighthouse Studio Help: CBC Questionnaires and Design Strategy [Internet]. 2020 [cited 2020 Jul 28]. Available from: https://legacy.sawtoothsoftware.com/help/lighthouse-studio/manual/.

[2] Orme BK. Fine-Tuning CBC and Adaptive CBC Questionnaires (2009) [Internet]. [cited 2020 Jul 28]. Available from: https://sawtoothsoftware.com/resources/technical-papers/fine-tuning-cbc-and-adaptive-cbc-questionnaires-2009.
