# Supplementary material for: SARS-CoV-2 Testing Service Preferences of Adults in the United States: Discrete Choice Experiment
Source: JMIR Public Health Surveill. 2020 Dec 31;6(4):e25546. doi: 10.2196/25546 (PMC7781587; doi:10.2196/25546)
Supplement: Multimedia Appendix 3 [file publichealth_v6i4e25546_app3.docx]

| **Table S1. Average relative attribute importance for SARS-CoV-2 testing features** | | | |
| --- | --- | --- | --- |
| **Attribute** | **Relative importance** | **Lower  95% CI** | **Upper  95% CI** |
|  |  |  |  |
| Test | 28.3% | 28.1% | 28.6% |
| Specimen type | 26.2% | 26.0% | 26.5% |
| Venue | 15.0% | 14.8% | 15.3% |
| Results turnaround time | 30.4% | 30.1% | 30.7% |
| *N=4,793, September 8, 2020.*  *Analyzed using Sawtooth Lighthouse Studio 9.8.1.* | | | |

| **Table S2. Part-worth utilities for SARS-CoV-2 testing features** | | | | |
| --- | --- | --- | --- | --- |
| **Attributes & Levels** | | **Part-worth utility**^a^ | **Lower  95% CI** | **Upper  95% CI** |
|  | |  |  |  |
| **Test** | |  |  |  |
|  | Serology | -37.1 | -37.9 | -36.3 |
|  | PCR | -24.6 | -25.3 | -24.0 |
|  | Both tests | 61.7 | 61.0 | 62.4 |
| **Specimen type** | |  |  |  |
|  | Finger prick | 16.3 | 15.7 | 16.8 |
|  | Blood draw | -19.4 | -20.1 | -18.8 |
|  | Cheek | 24.9 | 24.5 | 25.3 |
|  | Spit | 25.1 | 24.5 | 25.6 |
|  | Nasal shallow | 3.4 | 2.9 | 3.8 |
|  | NP swab | -63.4 | -64.3 | -62.5 |
|  | Urine | 13.2 | 12.7 | 13.7 |
| **Venue** | |  |  |  |
|  | Home collection, receiving kit in mail & returning kit in mail | 13.3 | 12.5 | 14.2 |
|  | Home collection, receiving kit in mail & returning to a collection site | 9.6 | 8.9 | 10.3 |
|  | Doctor's office or urgent care clinic | -14.4 | -15.0 | -13.8 |
|  | Walk-in community testing site | -16.8 | -17.5 | -16.2 |
|  | Drive-through community testing site | 4.8 | 4.3 | 5.3 |
|  | Pharmacy | 3.5 | 3.1 | 3.9 |
| **Results turnaround time** | |  |  |  |
|  | Immediate | 56.5 | 55.8 | 57.2 |
|  | Same day | 35.3 | 34.8 | 35.8 |
|  | 48 hours | 6.7 | 6.4 | 7.0 |
|  | 5 days | -37.8 | -38.3 | -37.2 |
|  | GT 5 days | -60.7 | -61.6 | -59.9 |
| **None** | | -228.7 | -231.0 | -226.4 |
| *^a^Part-worth utilities are zero-centered.*  *N=4,793, September 8, 2020.*  *Analyzed using Sawtooth Lighthouse Studio 9.8.1.* | | | | |
